# Artificial intelligence-driven circRNA vaccine development: multimodal collaborative optimization and a new paradigm for biomedical applications

**DOI:** 10.1093/bib/bbaf263

**Published:** 2025-06-08

**Authors:** Yan Zhao, Huaiyu Wang

**Affiliations:** Department of Hematology, The First Affiliated Hospital of Xi’an Jiaotong University, 277 West Yanta Road, Xi’an, Shaanxi 710061, P.R. China; Department of Hematology, The First Affiliated Hospital of Xi’an Jiaotong University, 277 West Yanta Road, Xi’an, Shaanxi 710061, P.R. China

**Keywords:** circRNA vaccine, artificial intelligence, deep learning, generative AI, bioinformatics AI

## Abstract

Circular RNA (circRNA) vaccines have emerged as a groundbreaking innovation in infectious disease prevention and cancer immunotherapy, offering superior stability and reduced immunogenicity compared to conventional linear messenger RNA (mRNA) vaccines. While linear mRNA vaccines are prone to degradation and can trigger strong innate immune responses, covalently closed circRNA vaccines leverage their unique circular structure to enhance molecular stability and minimize innate immune activation, positioning them as a next-generation platform for vaccine development. Artificial intelligence (AI) is revolutionizing circRNA vaccine design and optimization. Deep learning models, such as convolutional neural networks (CNNs) and Transformers, integrate multi-omics data to refine antigen prediction, RNA secondary structure modeling, and lipid nanoparticle delivery system formulation, surpassing traditional bioinformatics approaches in both accuracy and efficiency. While AI-driven bioinformatics enhances antigen screening and delivery system modeling, generative AI accelerates literature synthesis and experimental planning—though the risk of fabricated references and limited biological interpretability hinders its reliability. Despite these advancements, challenges such as the “black-box” nature of AI algorithms, unreliable literature retrieval, and insufficient integration of biological mechanisms underscore the necessity for a hybrid “AI-traditional-experimental” paradigm. This approach integrates explainable AI frameworks, multi-omics validation, and ethical oversight to ensure clinical translatability. Future research should prioritize mechanism-driven AI models, real-time experimental feedback, and rigorous ethical standards to fully unlock the potential of circRNA vaccines in precision oncology and global health.

## Introduction

### Advancements and prospects of mRNA and circRNA vaccine technologies

Messenger RNA (mRNA) vaccines have revolutionized immunotherapy, offering rapid development and scalable production [1–3]. Their success during the coronavirus disease 2019 (COVID-19) pandemic, with Pfizer-BioNTech (BNT162b2) and Moderna (mRNA-1273) vaccines demonstrating 94%–95% efficacy within a year of development, highlights their transformative potential [4–6]. Moreover, post-market surveillance confirms low rates of severe adverse events, reinforcing their safety profile [7]. These advancements establish mRNA vaccines as a promising platform for cancer immunotherapy and other precision medicine applications [8–10].

Unlike conventional inactivated or subunit vaccines that introduce pre-formed antigens, mRNA vaccines encode antigens for *in vivo* expression, eliciting both *CD8^+^* T cell-mediated cytotoxic immunity [*via* Major Histocompatibility Complex Class I (MHC-I)] and B cell-driven humoral responses [11–14]. To enhance immunostimulation, mRNA sequences can be engineered to co-activate STING and TLR3/7/8 pathways, promoting dendritic cell (DC) maturation (*CD80/86* expression) and pro-inflammatory cytokine secretion (*IL-12, IFN-α*). This improves antigen presentation efficiency and strengthens the *T-helper 1 (Th1)* immune response [15–17]. Beyond immunogenicity, mRNA vaccines offer significant manufacturing advantages. Unlike traditional vaccines requiring cell culture and protein purification, mRNA vaccines can be synthesized *via* a simple *in vitro* transcription (IVT) reaction, dramatically reducing production time and scalability constraints [18]. Their modular design also enables rapid customization, allowing swift adaptation to emerging viral variants and personalized cancer immunotherapy [19].

The current global development of mRNA vaccines relies on a linear molecular architecture, consisting of a 5′ cap, untranslated regions (UTRs), an open reading frame (ORF), and a 3′ poly(A) tail, with antigen expression initiated *via* cap-dependent translation [20, 21]. However, linear mRNA is highly susceptible to exonuclease degradation. To enhance stability, researchers have introduced N1-methyl-pseudouridine (1mΨ) and other nucleoside modifications, significantly increasing RNA half-life [22]. Additionally, lipid nanoparticle (LNP) formulation adjustments, such as reducing polyethylene glycol (PEG) content, may improve cellular uptake and endosomal escape. However, careful optimization is required to balance circulation stability and delivery efficiency [23, 24]. As a more stable alternative, circular RNA (circRNA) exhibits a 10-fold increase in nuclease resistance compared to linear mRNA, largely due to its topologically closed structure [25, 26]. During clinical translation, microfluidic-based manufacturing must tightly regulate particle size and minimize residual double-stranded RNA (dsRNA) contamination [27, 28] Furthermore, ultra-stable 5′-UTR elements, identified through directed evolution, can increase protein expression up to 34-fold, offering a crucial advantage in combating viral mutations and tumor heterogeneity [29–31].

circRNA represents a distinct RNA class, formed through back-splicing, with enhanced structural stability [32]. Lacking free 5′ and 3′ termini, circRNA is not only resistant to exonuclease degradation but also evades immune recognition by pattern recognition receptors (PRRs), making it an attractive candidate for nucleic acid drug delivery [33]. In the biomedical field, circRNA is emerging as a promising vector for gene therapy and vaccine antigen expression, owing to its immune evasion capabilities and metabolic stability [34–36].

Recent advances in circRNA therapeutics have expanded their potential across multiple diseases, including cancer, cardiovascular and neurodegenerative disorders, autoimmune diseases, ocular conditions, and viral infections. In oncology, circRNAs modulate tumor proliferation, metastasis, and therapeutic resistance through mechanisms such as miRNA sponging, protein scaffolding, and translation of functional peptides, positioning them as candidate biomarkers and therapeutic targets [37]. In cardiovascular disease, their inherent stability renders them promising vectors for gene therapy, particularly in pathologies such as myocardial hypertrophy and atherosclerosis [38]. In neurodegenerative diseases, including Alzheimer’s and Parkinson’s, dysregulated circRNA expression has been implicated in synaptic dysfunction and neuroinflammation, although efficient delivery across the blood–brain barrier remains a key limitation [39]. Within autoimmune and inflammatory contexts—such as rheumatoid arthritis and pulmonary fibrosis—circRNAs contribute to the regulation of immune signaling and tissue remodeling [40]. In ocular disorders, including diabetic retinopathy and glaucoma, circRNAs are involved in angiogenic processes, while in osteoarthritis, they modulate disease progression via chondrocyte-associated miRNA networks [41]. Moreover, the structural stability and extended translational potential of circRNAs have prompted their exploration as novel vaccine platforms, exemplified by their integration into emerging RNA vaccine technologies against pathogens such as Severe acute respiratory syndrome coronavirus 2 (SARS-CoV-2) [42]. However, technical and translational barriers remain, requiring further optimization in synthesis, delivery, and large-scale production to fully unlock its therapeutic potential.

### Synthesis, delivery, and immune regulation of circRNA

CircRNA technology encounters multifaceted challenges in biosynthesis and delivery [43]. In the synthesis process, conventional IVT systems are limited by inefficient circularization [44] and intron retention, which aberrantly activate the TLR3/RIG-I signaling pathways [45]. To address these issues, topoisomerase-mediated covalent closure strategies must be developed to enhance circularization efficiency. Furthermore, a robust quality control (QC) system driven by ultra-high-performance liquid chromatography (UHPLC) is essential to monitor byproducts such as dsRNA [46].

Regarding delivery, LNPs exhibit lower encapsulation efficiency for circRNA than linear mRNA, potentially due to the terminal structures of linear mRNA facilitating electrostatic interactions with LNP cationic lipids, whereas circRNA’s circular structure exhibits weaker electrostatic affinity. Additionally, the circularization process may generate linear RNA or dsRNA byproducts, which could interfere with LNP encapsulation, further reducing the proportion of effective payloads [47]. Traditional LNP formulations, such as those incorporating the ionizable lipid ALC-0315, were optimized for linear mRNA and are thus suboptimal for circRNA. However, novel lipid formulations provide promising alternatives: e.g. LNPs formulated with the ionizable lipid U-105 demonstrate improved particle size uniformity and stability in dynamic light scattering and transmission electron microscopy analyses, thereby enhancing circRNA loading capacity [48]. Moreover, customized LNP platforms, such as H1L1A1B3 LNPs, have increased *in vivo* circRNA delivery efficiency four-fold by optimizing lipid composition (ionizable lipid:cholesterol:DSPC:PEG = 50:38.5:10:1.5) and surface PEG density [49].

In the context of immune regulation in circRNA vaccines, although the circular topology theoretically mitigates TLR7/8-mediated innate immune activation [50], residual byproducts generated during IVT, such as dsRNA, may not be eliminated. These dsRNA contaminants can activate type I interferon (IFN) responses *via* non-TLR pathways, such as RIG-I-like receptors (e.g*.* MDA5), leading to the induction of pro-inflammatory cytokines such as IFN-γ [51]. Additionally, while LNPs serve as efficient delivery vehicles for circRNA, their cationic lipid components may inadvertently stimulate intracellular PRRs such as the STING pathway, further enhancing IFN-γ secretion [49]. If the circRNA encodes an antigen—such as a tumor-associated or viral antigen—its sustained expression *in vivo* may induce MHC-I cross-presentation, triggering CD8^+^ T cell activation and subsequent IFN-γ release [52]. To mitigate these immunogenic risks and enhance safety, it is crucial to optimize purification strategies, such as high-performance liquid chromatography (HPLC) or RNase R digestion, to efficiently remove residual dsRNA contaminants [51]. Additionally, refining circularization techniques to minimize byproducts, optimizing LNP formulations [53], and incorporating miRNA binding sites in sequence design can facilitate tissue-specific expression, thereby reducing off-target immune responses and improving overall biocompatibility [51].

From a functional perspective, circRNA exhibits a unique molecular regulatory network. Single-molecule imaging has demonstrated its ability to simultaneously sequester multiple miRNAs (e.g*.* miR-21/155), establishing a competitive endogenous RNA (ceRNA) network that modulates epithelial-mesenchymal transition (EMT) and cell cycle progression [51]. Despite databases such as circBase cataloging certain functional circRNAs, their coverage remains below 10%, limiting their applicability in complex diseases such as cancer and diabetic foot ulcers. For instance, a circRNA-based therapeutic (e.g. VEGF-A-encoding circRNA/LNP formulation) has been shown to accelerate wound healing following a single administration. Nevertheless, a more comprehensive analysis of its regulatory mechanisms in angiogenesis and inflammation resolution is required [48]. Cryo-electron microscopy structural analyses have revealed that uneven stress distribution at circular junctions may lead to structural fragility, necessitating the optimization of back-splicing site design to preserve ORF integrity. A notable example is the use of naturally derived viral internal ribosome entry sites (IRES), such as the hepatitis C virus (HCV) 5' UTR, in circRNA vaccine constructs, which, upon LNP encapsulation, exhibit sustained antigen expression and robust immune activation, offering a model for structural refinement [50].

### Applications and challenges of artificial intelligence in circRNA research and immunotherapy

In recent years, artificial intelligence (AI) has emerged as a transformative force in scientific research, driving advancements in pattern recognition, data processing, and problem-solving across multiple disciplines. Within molecular and cellular biology, AI has evolved from traditional statistical analysis to sophisticated machine learning and deep learning approaches, significantly enhancing research efficiency and providing novel insights into unresolved biological questions [54]. Conventional experimental design often relies on researcher intuition and hypothesis-driven approaches, which, while informative, have inherent limitations in precision and efficiency. AI-driven methodologies, leveraging data-intensive analytics, offer a more intelligent and efficient framework for experimental design, particularly in optimizing sequence design, stability, and translational efficiency for mRNA vaccine development [55].

The Generative Pre-trained Transformer (GPT), a large language model (LLM) developed by OpenAI, has demonstrated remarkable capabilities in natural language processing (NLP) and multimodal tasks through extensive pretraining and fine-tuning. Since the release of GPT-1 and GPT-3, the model has exhibited exceptional performance across various applications, including dialogue systems and content generation [56, 57]. While ChatGPT has shown outstanding proficiency in language generation and knowledge-based querying, its reliability and applicability in clinical environments and molecular biology research require further validation [58, 59].

Similar to GPT, DeepSeek is an emerging AI model specializing in deep learning and data mining, designed for high-precision predictive modeling and decision support [60]. DeepSeek excels in identifying complex data patterns and conducting in-depth analyses, making it highly applicable to domains such as finance, healthcare, and retail. In molecular biology, DeepSeek’s deep neural network architecture, which integrates self-supervised and reinforcement learning techniques, enables the detection of hidden relationships within large datasets, thereby facilitating disease diagnosis, drug discovery, and personalized medicine. Its analytical capabilities stem from reinforcement learning mechanisms, encompassing data classification, trend prediction, and anomaly detection [60].

Since its launch on 5 January 2024, DeepSeek has rapidly gained prominence, with a growing user base and multiple iterations enhancing its capabilities. The initial versions, DeepSeek LLM and DeepSeek-Coder, focused on fundamental data analysis and pattern recognition, while subsequent iterations—DeepSeek-VL, DeepSeek V2, and DeepSeek V3—incorporated more advanced algorithms and expanded datasets, further refining accuracy and efficiency. Notably, DeepSeek-V3, based on the Mixture of Experts (MoE) architecture, features a parameter count of 671 billion, with only 37 billion of them activated per token, enabling high-efficiency inference and cost-effective training through innovations such as Multi-head Latent Attention (MLA), optimized MoE structures (DeepSeekMoE), and zero-loss auxiliary load balancing. Shortly after its release, DeepSeek-R1-Zero, a first-generation deep inference model, was introduced, undergoing multi-stage optimization through supervised fine-tuning (SFT) and reinforcement learning to enhance reasoning and output quality. DeepSeek-R1 series is available under the MIT license, allowing unrestricted use, modification, and commercialization. This open-access strategy has accelerated AI innovation, enabling local deployment without reliance on proprietary platforms like OpenAI [60].

DeepSeek has achieved reasoning capabilities comparable to leading AI models such as those developed by OpenAI, while also contributing to advancements across the broader AI landscape. It demonstrates unique potential in clinical workflows, medical education, and research, with its open-source architecture enabling continuous integration of the latest scientific reasoning and evidence-based medicine. A comparative study evaluating ChatGPT-1 and DeepSeek-R1 in pediatric clinical decision support assessed their accuracy, reasoning logic, and practical application using a MedQA dataset of 500 multiple-choice questions [61]. The results showed that ChatGPT-1 achieved a higher accuracy rate (92.8%), excelling in complex clinical reasoning, whereas DeepSeek-R1, despite a slightly lower accuracy (87.0%), leveraged its open-source nature to support self-improvement and iterative refinement, making it more suitable for resource-limited healthcare settings. Moreover, DeepSeek’s offline deployment capabilities offer significant value to medical institutions by enabling local model execution, thereby reducing reliance on internet connectivity and enhancing patient data privacy protection [60, 62].

Leveraging its technological advantages, DeepSeek’s multimodal data processing capabilities hold significant promise for molecular and cellular biology, particularly for integrating cross-omics data and enabling dynamic modeling in genomics, proteomics, and metabolomics [63]. However, despite its strengths, DeepSeek faces several critical challenges in real-world applications, including data dependency, training bias, and algorithmic opacity, which limit its transparency and interpretability. Additionally, its clinical applicability remains uncertain due to the lack of real-time validation in clinical settings. Given the rapid evolution of AI research, current studies on DeepSeek’s clinical applications primarily rely on preprints or non-peer-reviewed materials, underscoring the necessity of large-scale randomized controlled trials to confirm its regulatory compliance and clinical utility.

While generative AI excels in NLP, bioinformatics-specific AI tools provide unique advantages for molecular analysis by leveraging curated biological datasets and biophysical constraints. Key advances include molecular interaction predictors such as AlphaFold for RNA-protein structures, functional annotation models such as DeepBind for epitope screening, and genome-scale systems like Evo 2 for synthetic biology applications. These tools complement generative AI by offering experimentally validated, multiscale frameworks from atomic-level predictions to whole-genome engineering, enabling both mechanistic depth and systemic breadth that are essential for circRNA vaccine development. Table 1 shows the technical specifications of bioinformatics AI tools.

**Table 1:** Bioinformatics AI technical specifications

Tool	Type	Architecture/Algorithm	Training data	Key parameters	Application	License
AGILE	Ionizable lipid design	GNN + combinatorial chemistry	60K virtual lipids +1.2K experimental LNPs (HeLa/RAW 264.7)	pKa = 6.5–7.0, log*P* = 2–5, Tail1 = C10–18	AI-guided LNP optimization for mRNA delivery	Proprietary
AlphaFold 3	Protein-RNA structure prediction	Geometric Diffusion + Evoformer (48 layers)	PDB + AlphaFold DB (2.2M structures, incl. multimer data)	Evoformer_layers = 48	circRNA-protein interaction	Academic
BERTome	Transcriptome language model	BERT (12 layers) + CNN	UniProt sequences (250M) + PubMed abstracts	seq_len = 512, masked_lm_loss	Protein function annotation	Open-source
ChatGPT	Conversational AI	Transformer (175B params, RLHF fine-tuned)	OpenAI corpus (Common Crawl, books, code)	context_window = 128 k, max_tokens = 4096	Protocol design, literature Q&A	Freemium
CircDesign	circRNA optimization	Transformer + dynamic programming	50K circRNA sequences	GC = 40%–60%, IRES efficiency>0.8, MFE < −300 kcal/mol	circRNA vaccine/therapeutics design	Open-source
DeepAlign	Sequence alignment	CNN + transformer hybrid	100K spliced RNA-Seq reads (ENCODE)	k-mer = 21, min_alignment_score = 0.8	circRNA splice variant analysis	Open-source
DeepBind	RNA/DNA binding prediction	CNN (4 convolutional layers)	ENCODE RBP binding sites (500K peaks)	motif_len = 8–11, score_threshold = 0.05	circRNA-protein binding sites	Open-source
DeepImmuno	Immune response modeling	CNN + LSTM + ODEs	IEDB epitopes (500K) + immune activation assays	dropout = 0.2, LR = 3e−5	Immunotherapy efficacy prediction	Commercial
DeepSeek-R1	RNA sequence optimization	Transformer (12 layers) + dynamic programming	Internal IVT data (50K sequences) + tRNA databases	LR = 1e−5, batch = 32, epochs = 50	circRNA vaccine sequence design	Proprietary
DeepVariant	Variant calling	CNN (Inception-v3)	GIAB benchmark v4.2 (3000 genomes)	base_quality = 20, min_count = 2	circRNA mutation detection	Open-source
Elicit	Literature retrieval	GPT-4 fine-tuned	10 M PubMed/arXiv full-text articles	top_k = 50, max_tokens = 4096	Protocol summarization	Freemium
Evo 2	Generative genome design	StripedHyena-2 (7B params)	9.3 trillion bp (Bacteria/Archaea/Eukaryotes)	context_window = 1 M	circRNA-host genome optimization	Proprietary
FastQ Screen	NGS contamination check	Bowtie2-based alignment	15 reference genomes (hg38/mm10 + contaminants)	mismatch = 1, seed_len = 20	Sequencing data QC	Open-source
Geneformer	Transcriptome analysis	BERT (12 layers, 768 hidden dim)	CELLxGENE Census (23.6 M human scRNA-Seq cells)	heads = 12, context = 2048	Cell-type-specific RNA analysis	Commercial
GraphAligner	Graph-based alignment	GNN	100K spliced RNA-Seq reads (SRA long-read datasets)	*k* = 15/19, band_width = 100	Long-read genome alignment	Open-source
LinearDesign	mRNA sequence optimization	Dynamic programming + ML	Internal lab data (50K sequences) + cAIdb	CAI > 0.8, GC_content = 40%–60%, U-content < 25%	circRNA vaccine design	Open-source
MAQC-III (SEQC)	NGS quality control	Cross-platform RNA-Seq alignment	Two human reference RNAs (UHRR, Brain)	DEG threshold: |logFC| > 2, F1 > 0.95	Clinical RNA-Seq standardization	Open-source
NetMHCpan-4.1	MHC–peptide binding prediction	ANN	IEDB + MassIVE (850K peptides, 170 MHC-I alleles)	strong_binders: IC50 ≤ 50 nM, weak≤500 nM	circRNA vaccine epitope screening	Academic
NVIDIA BioNeMo	Biomolecular foundation models	Hybrid (transformers/VAEs/ANNs)	UniRef50 + ChEMBL (10 M molecules)	LR = 2e−5, batch = 32	Drug-target interaction prediction	Proprietary
NVIDIA Clara	Healthcare AI	CNN + NLP	TCIA + RadImage (1 M CT/MRI/X-ray images)	max_epochs = 50, LR = 1e−4	Medical image analysis	Proprietary
PyTorch	Deep learning framework	Dynamic computation graph	N/A (framework)	CUDA acceleration, autograd	General AI/ML development	Open-source
TensorFlow	LNP optimization	CNN + LSTM	10K LNP formulations (industry partners)	lipid_ratio_tolerance = ±5%	circRNA delivery system design	Open-source

Abbreviations: AGILE, AI-guided Ionizable Lipid Engineering; AlphaFold 3, AlphaFold 3 (protein-RNA structure prediction tool); BERTome, Bidirectional Encoder Representations from Transformers for Transcriptomics; ChatGPT, Chat Generative Pre-trained Transformer; CircDesign, Circular RNA Design; DeepAlign, Deep Sequence Alignment; DeepBind, Deep Binding Prediction; DeepImmuno, Deep Immune Response Modeling; DeepSeek-R1, DeepSeek RNA Sequence Optimization; DeepVariant, Deep Learning-based Variant Calling; Elicit, Literature Retrieval via AI; Evo 2, Evolutionary Genome Design 2; FastQ Screen, FastQ Quality Control Screening; Geneformer, Gene Expression Transformer; GraphAligner, Graph-based RNA Alignment; LinearDesign, Linear mRNA Sequence Optimization; MAQC-III (SEQC), MicroArray Quality Control III (Standardization and Evaluation Consortium); NetMHCpan-4.1, Network MHC Pan-specific Peptide Binding; NVIDIA BioNeMo, BioMolecular Neural Network Model; NVIDIA Clara, Clara Healthcare AI Platform; PyTorch, Python-based Tensors and Optimization Framework; TensorFlow, Tensor Flow Machine Learning Framework; GNN, Graph Neural Network; CNN, Convolutional Neural Network; BERT, Bidirectional Encoder Representations from Transformers; Transformer, Self-Attention Transformer; RLHF, Reinforcement Learning from Human Feedback; Dynamic Programming, Dynamic Programming Algorithm; StripedHyena-2, StripedHyena 2.0 architecture; Bowtie2, Bowtie 2; Inception-v3, Inception v3 CNN; ANN, Artificial Neural Network; VAEs, Variational Autoencoders; CUDA, Compute Unified Device Architecture; autograd, Automatic Differentiation; UniProt, Universal Protein Resource; PubMed, PubMed Central; Common Crawl, Web-scale Common Crawl Corpus; ENCODE, Encyclopedia of DNA Elements; SRA, Sequence Read Archive; IEDB, Immune Epitope Database; MassIVE, Mass Spectrometry Imaging Virtual Experiment; UniRef50, UniProt Reference Clusters 50; ChEMBL, Chemical Biology Database; TCIA, The Cancer Imaging Archive; pKa, Acid Dissociation Constant; logP, Octanol–Water Partition Coefficient; Tail1, Lipid Tail Length; IVT, *In Vitro* Transcription; tRNA, Transfer RNA; LR, Learning Rate; CAI, Codon Adaptation Index; GC, Guanine-Cytosine Content; U, Uracil Content; DEG, Differentially Expressed Gene; logFC, Log Fold Change; F1, F1 Score; IC50, Half-Inhibitory Concentration; MHC-I, Major Histocompatibility Complex Class I; scRNA-Seq, Single-Cell RNA Sequencing; QC, Quality Control; Proprietary, Proprietary Software; Academic, Academic License; Open-source, Open-source License (e.g. MIT/GPL); Freemium, Freemium Model (free tier + premium features); Commercial, Commercial License; N/A, Not Applicable; LNP, Lipid Nanoparticle; mRNA, Messenger RNA; circRNA, Circular RNA; MFE, Minimum Free Energy; kcal/mol, Kilocalories per Mole.

**Figure 1:**
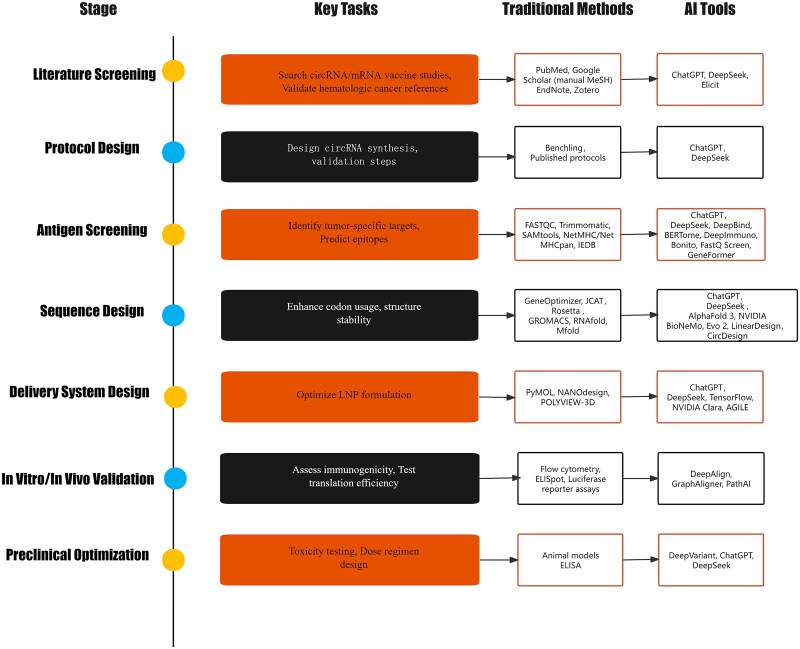
Comparative workflow of circRNA vaccine development using traditional methods and AI-driven tools.

This study aims to analyze the application of generative AI technologies, exemplified by ChatGPT and DeepSeek, as well as deep learning models trained on biological data, in circRNA vaccine research. Specifically, it explores the potential of AI-driven algorithms in circRNA sequence design, structural inference, and vaccine development, highlighting their role in optimizing therapeutic efficacy and translational applications. The overall workflow of our AI-assisted circRNA vaccine design pipeline is illustrated in Fig. 1.

## Methods and Results

### A research search on the progress of CircRNA vaccines

Generative AI has exhibited outstanding capabilities in processing and analyzing medical literature, thereby enabling the comprehensive integration of global scientific advancements. This technology facilitates real-time tracking of both basic and clinical research trends, offering researchers an efficient tool for dynamic data retrieval. By constructing intelligent knowledge graphs, AI systematically deciphers the differential expression patterns and regulatory networks of circRNA in tumors, dynamically linking these molecular characteristics to clinical staging, drug resistance, and prognosis. This enables the identification of tumor-specific circRNA targets and the development of precision therapeutic strategies, accelerating the transition from fundamental research to clinical applications.

To evaluate the performance of ChatGPT and DeepSeek in retrieving literature related to mRNA vaccines and hematologic malignancies, we used the following standardized prompt: “Please provide Vancouver-style references on mRNA vaccines and hematologic malignancies, along with their links.” The retrieved results were assessed for existence, relevance, and accuracy, including verification of titles, authors, journal names, publication years, DOI numbers, and access links. Literature validation was performed through manual cross-verification using Google Scholar and PubMed, with the latter employing the following comprehensive MeSH term strategy as a baseline: ((((((((((leukemia[Title/Abstract]) OR (lymphoma[Title/Abstract])) OR (multiple myeloma[Title/Abstract])) OR (myelodysplastic syndrome[Title/Abstract])) OR (myeloproliferative neoplasms[Title/Abstract])) OR (Hodgkin's disease[Title/Abstract])) OR (non-Hodgkin lymphoma[Title/Abstract])) OR (acute myeloid leukemia[Title/Abstract])) OR (chronic lymphocytic leukemia[Title/Abstract])) OR (((((((((((((((((((((((Hematologic Neoplasm[Title/Abstract]) OR (Hematologic Neoplasms[Title/Abstract])) OR (Hematologic Malignancies[Title/Abstract])) OR (Hematologic Malignancy[Title/Abstract])) OR (Hematological Malignancies[Title/Abstract])) OR (Hematological Malignancy[Title/Abstract])) OR (Malignancy, Hematological[Title/Abstract])) OR (Hematological Neoplasms[Title/Abstract])) OR (Hematological Neoplasm[Title/Abstract])) OR (Neoplasm, Hematological[Title/Abstract])) OR (Malignancies, Hematologic[Title/Abstract])) OR (Malignancy, Hematologic[Title/Abstract])) OR (Blood Cancer[Title/Abstract])) OR (Blood Cancers[Title/Abstract])) OR (Cancer, Blood[Title/Abstract])) OR (Neoplasms, Hematologic[Title/Abstract])) OR (Hematopoietic Neoplasms[Title/Abstract])) OR (Hematopoietic Neoplasm[Title/Abstract])) OR (Neoplasm, Hematopoietic[Title/Abstract])) OR (Neoplasms, Hematopoietic[Title/Abstract])) OR (Hematopoietic Malignancies[Title/Abstract])) OR (Hematopoietic Malignancy[Title/Abstract])) OR (Malignancy, Hematopoietic[Title/Abstract]))) AND (((((((((((((((((mRNA Vaccine[Title/Abstract]) OR (Vaccine, mRNA[Title/Abstract])) OR (Messenger RNA Vaccines[Title/Abstract])) OR (RNA Vaccines[Title/Abstract])) OR (Vaccines, RNA[Title/Abstract])) OR (Naked RNA Vaccines[Title/Abstract])) OR (RNA Vaccine[Title/Abstract])) OR (Vaccine, RNA[Title/Abstract])) OR (Self-Amplifying RNA Vaccine[Title/Abstract])) OR (RNA Vaccine, Self-Amplifying[Title/Abstract])) OR (Self Amplifying RNA Vaccine[Title/Abstract])) OR (Vaccine, Self-Amplifying RNA[Title/Abstract])) OR (saRNA Vaccine[Title/Abstract])) OR (Vaccine, saRNA[Title/Abstract])) OR (Trans Amplifying RNA Vaccine[Title/Abstract])) OR (taRNA Vaccine[Title/Abstract])) OR (Vaccine, taRNA[Title/Abstract])). This search yielded a total of 121 articles, which served as the benchmark dataset. Upon submitting the prompt to ChatGPT and DeepSeek, neither AI model was able to generate a complete and accurate reference list or specify the number of retrieved articles. ChatGPT generated the following search strategy in PubMed: (``mRNA vaccine''[MeSH Terms] OR ``mRNA vaccine''[All Fields] OR ``COVID-19 vaccine''[MeSH Terms] OR ``COVID-19 vaccine''[All Fields]) AND (``hematologic malignancy''[MeSH Terms] OR ``hematologic malignancy''[All Fields] OR ``blood cancer''[All Fields] OR ``hematologic cancer''[All Fields] OR ``leukemia''[MeSH Terms] OR ``lymphoma''[MeSH Terms] OR ``myeloma''[MeSH Terms]). After searching, 117 references were retrieved; however, 17.1% were unrelated to mRNA vaccines, instead focusing primarily on COVID-19 vaccines. Among the five specific references directly provided by ChatGPT, four had matching DOIs and links, yet their titles and DOIs did not correspond accurately. Furthermore, one DOI and four titles were entirely fabricated, and the author names were also deemed unreliable. In addition, one reference was entirely irrelevant to the search criteria, resulting in an effective reference accuracy rate of only 80%. DeepSeek utilized the following combined search query in PubMed: ("mRNA vaccine" OR "RNA vaccine") AND ("hematologic malignancy" OR "blood cancer" OR "leukemia" OR "lymphoma" OR "multiple myeloma"), retrieving 99 references in total. However, among the seven specific references provided by DeepSeek, all provided titles were fabricated. While four references had matching DOIs and links, only one article was relevant to the search topic, resulting in a reference accuracy rate of only 14.3%.

In summary, both ChatGPT and DeepSeek failed to fully incorporate MeSH terms and Entry Terms, which led to limited literature coverage. ChatGPT exhibited issues with fabricated DOIs (20%), mismatched titles (80%), and irrelevant content (20%), while DeepSeek had a title fabrication rate as high as 85.7%, with only 14.3% of the retrieved references being relevant. Therefore, in rigorous academic contexts such as meta-analyses, AI-driven tools cannot yet replace traditional literature retrieval methods.

### CircRNA-related protocol design

Generative AI has emerged as a powerful tool for automating circRNA research workflows, spanning synthesis, modification, functional validation, and both *in vitro* and *in vivo* applications. By integrating research objectives and background information, AI can construct comprehensive experimental frameworks, detailing methodological steps, reagent specifications, material inventories, and expected outcomes, thereby significantly expediting protocol development.

In the context of *in vitro* circRNA synthesis, AI-generated protocols typically incorporate: (i) circRNA synthesis and modification, including linear RNA IVT, RNA purification, and chemical modifications; (ii) functional validation, involving quantitative polymerase chain reaction (qPCR), Northern blot analysis, protein-binding assays, cellular functional studies, and *in vivo* validation. With further refinement, AI can suggest experimental parameters, such as annealing temperature gradients and antibody concentrations. However, certain AI-generated steps diverge from standard protocols, and without experimental validation, their reliability remains uncertain. For instance, when querying ChatGPT-4o and DeepSeek-R1 with the prompt: “Provide a semi-quantitative PCR protocol using Taq polymerase,” a comparison with peer-reviewed literature [64] revealed discrepancies in Taq polymerase concentrations and a lack of electrophoretic evidence supporting amplification specificity (Table 2).

The credibility of literature-based protocols stems from peer review and empirical validation, often supported by electrophoretic images and quantitative data that ensure reproducibility. By contrast, while AI-generated protocols allow greater flexibility in parameter settings, their lack of iterative experimental verification introduces potential methodological risks. Nevertheless, AI-driven NLP techniques facilitate systematic workflow generation, integrating experimental steps, reagent recommendations, and anticipated results, thereby reducing protocol design time from 8–12 h to as little as 10 min. This efficiency is especially beneficial for preliminary gene expression screening and PCR training. However, human expertise remains indispensable in formal experimentation. AI cannot supplant researchers' domain knowledge and experimental intuition; therefore, scientists must critically refine AI-generated protocols to ensure their practical applicability and scientific rigor.

**Table 2:** Comparison of literature-validated, ChatGPT-generated, and DeepSeek-generated PCR protocols

Comparison	Literature-validated PCR protocol	ChatGPT-provided PCR protocol	DeepSeek-provided PCR protocol	Main differences
Total volume	25 μl	25 μl	25 μl	No difference
Taq buffer (10×)	2.5 μl	2.5 μl	2.5 μl	No difference
dNTP final concentration	0.2 mM (10 mM dNTPs 0.5 μl)	2.5 mM (1 mM each)	2.5 mM (2.0 μl)	ChatGPT and DeepSeek protocols have a higher dNTP concentration, which may impact amplification specificity
Taq polymerase amount	1 μl (1 U)	0.5 μl (0.5 U)	0.2–0.5 μl (1–2.5 U)	ChatGPT uses less Taq polymerase, DeepSeek allows 0.2–0.5 μl (1–2.5 U) for optimization
MgCl₂ concentration	Not specified	1.5 mM (optional)	1.5–2.5 mM (adjustable)	DeepSeek provides an adjustable MgCl₂ range (1.5–2.5 mM), ChatGPT defaults to 1.5 mM
Template (cDNA amount)	5 μl (>10 ng)	1 μl (10–100 ng)	1–2 μl (adjustable based on concentration)	Literature protocol uses a higher template volume (5 μl), ChatGPT uses less (1 μl), DeepSeek allows flexibility (1–2 μl)
Primer amount	1 μl (10 μM)	1 μl (10 μM)	0.5 μl (10 μM)	DeepSeek uses a lower primer amount (0.5 μl versus 1 μl)
Water volume	14 μl	Adjusted to 25 μl	Adjusted to 25 μl	ChatGPT adjusts water volume based on other components
Reaction mix preparation	Prepare 20 μl Master Mix first, then add 5 μl cDNA	All components mixed at once	Target gene + reference gene amplified simultaneously	Literature protocol uses separate preparation to reduce error, ChatGPT mixes all at once, DeepSeek co-amplifies target and reference genes
Negative control (H₂O)	Contains 15 μl Master Mix +5 μl H₂O	Not explicitly mentioned	Contains NTC (No Template Control)	Literature and DeepSeek protocols emphasize negative controls, ChatGPT does not explicitly mention them
Denaturation temperature	95°C 2 min	94°C 2–5 min	95°C 3–5 min	Literature: 95°C for 2 min, ChatGPT: 94°C for 2–5 min, DeepSeek: 95°C for 3–5 min
Annealing temperature	60°C	55–65°C (based on primer design)	Tm-5°C	Literature: 60°C, ChatGPT: 55°C–65°C (broader range), DeepSeek: Tm-5°C (more flexible)
Extension temperature	72°C	72°C	72°C (1 kb/min)	No significant difference
Cycle number	30–35	25–35	25–35 (optimized)	Literature: 30–35, ChatGPT: 25–35, DeepSeek: 25–35 (needs optimization)
Final extension time	15 s	5 min	5–10 min	Literature: 15 s, ChatGPT: 5 min, DeepSeek: 5–10 min (better for long-fragment amplification)
Gel electrophoresis analysis	2% agarose gel	1.5%–2% agarose gel	1.5%–2% agarose gel (GelRed staining)	Literature: 2% gel, ChatGPT: 1.5%–2%, DeepSeek: 1.5%–2% (GelRed staining for higher sensitivity)
Quantification method	Band intensity observation	Gray value ratio calculation	ImageJ band intensity analysis	Literature relies on visual observation, ChatGPT and DeepSeek use grayscale analysis

Abbreviations: Taq, *Thermus aquaticus* DNA Polymerase; dNTP, Deoxyribonucleoside Triphosphate; cDNA, Complementary DNA; NTC, No Template Control; H₂O, Hydrogen Dihydrogen Oxide; MgCl₂, Magnesium Chloride; μl, Microliter; mM, Millimolar; U, Unit; ng, Nanogram; PCR, Polymerase Chain Reaction; Gel Electrophoresis, Gel Electrophoresis; ImageJ, Image Processing and Analysis in Java; Tm, Melting Temperature; bp, Base Pair.

### The antigen screening for circRNA vaccines

Accurate antigen screening for tumor vaccines relies heavily on high-quality multi-omics data. In recent years, the integration of generative AI models such as ChatGPT with classical bioinformatics tools like FASTQC, Trimmomatic, and SAMtools has facilitated the automation and standardization of end-to-end data analysis. This comprehensive approach encompasses QC, sequence alignment, variant detection, and gene expression analysis RNA sequencing (RNA-Seq). Additionally, AI-driven bioinformatics tools, including Bonito, FastQ Screen, and GeneFormer, have further optimized sequencing signal processing, data QC, noise reduction, alignment, and RNA-Seq analysis.

For instance, in RNA-Seq studies of hematologic malignancies, traditional tools such as Trimmomatic require manual parameter configuration to filter low-quality reads (e.g. Phred score < Q20), detect GC content anomalies, and remove adapter contamination. Researchers must continuously adjust sliding window sizes, quality thresholds (e.g*.* Q25 for a 4-bp window), and adapter removal criteria to ensure data accuracy [65, 66]. In contrast, AI-powered tools such as FastQ Screen and SEQC offer intelligent optimization of next-generation sequencing (NGS) data QC. FastQ Screen is not designed for predicting and trimming low-quality reads. Its primary function is to align sequencing reads against multiple reference genomes to ascertain the sample's origin and identify potential contamination [67]. The SEQC leverages large-scale, multi-platform datasets, such as B-cell lines, to train models capable of automatically identifying sequencing error patterns [68]. By integrating biological context, including known oncogenes and splice sites, it minimizes the erroneous removal of functionally relevant regions. The methodology employs the Maximum Rank Reproducibility (MaRR) approach, utilizing non-parametric statistical models to effectively control the false discovery rate (FDR) across batch effects [69, 70].

Building on these advancements, DeepSeek functions as a generative AI model capable of automatically parsing FASTQC reports and generating optimization recommendations. For example, when a decrease in 3′ end quality values is detected in Illumina sequencing data, DeepSeek might suggest adjusting Trimmomatic’s sliding window to 5 bp@Q30 for improved data filtering. Moreover, DeepSeek leverages Transformer models to predict and reconstruct missing fragments, correct mismatches, and enhance data integrity, thereby minimizing data loss and ensuring a more efficient and precise QC process.

Beyond QC, AI has substantially enhanced RNA-Seq alignment and variant detection. Traditional tools such as HISAT2, BWA, and Bowtie2 require manual configuration of parameters, including seed length, mismatch thresholds, and alignment limits. Poor-quality alignments with low mapping quality can compromise variant detection and downstream analyses, necessitating additional parameter tuning. In contrast, AI-driven tools like DeepAlign and GraphAligner streamline this process using deep learning. DeepAlign integrates graph neural networks (GNNs) and Transformer models to facilitate automatic parameter optimization, reducing the need for manual adjustments [71]. GraphAligner, leveraging GNNs, is particularly effective in structural variant detection and spliced alignment, enhancing alignment accuracy [72]. DeepSeek further expands the application of generative AI by dynamically adjusting alignment parameters based on FASTQC reports. By predicting and correcting low-confidence alignment regions, DeepSeek minimizes erroneous read deletions, making it particularly valuable for variant detection, structural variant analysis, and circRNA research.

Variant detection and annotation have also seen significant improvements with AI. Conventional tools such as SAMtools depend on fixed thresholds to identify SNPs and InDels, which can lead to high false-positive rate [73]. In contrast, AI-powered approaches like DeepVariant employ convolutional neural networks (CNNs) to improve detection accuracy [73]. When integrated with DeepSeek’s pathogenicity prediction capabilities and databases such as ClinVar and gnomAD, AI systems can automate variant annotation. For example, upon detecting NM_004119.3(FLT3):c.2028C > A (p.Asn676Lys), an AI system might output: "This mutation potentially impacts FLT3 protein function and is associated with acute myeloid leukemia (AML)." Compared to traditional methods, AI-based variant interpretation offers greater precision and contextual understanding.

The integration of multi-omics data is essential for identifying key epitopes, such as MHC-binding peptides and B/T cell epitopes that activate the immune system. In circRNA vaccine development, accurate antigen screening is essential to ensure both efficacy and safety. However, conventional tools such as NetMHC and immune epitope database and analysis resource (IEDB-AR) are constrained by data dependency and limited multi-omics integration [74, 75]. AI-driven deep learning models offer substantial advantages in this area. For instance, DeepBind employs CNNs to predict circRNA interactions with miRNAs or proteins, while BERTome, utilizing BERT-based LLMs, analyzes cancer genomic data to predict tumor-associated circRNA targets [76]. Evo2 models circRNA/mRNA mutation effects in cancer, simulating how RNA variants influence competing ceRNA networks and signaling pathways [77].

By integrating generative AI with LLMs and multi-omics data, researchers can generate automated, comprehensive tumor target reports. For example, when prompted to analyze differential circRNA expression in AML and identify potential targets associated with tumor invasion and metastasis, both DeepSeek and ChatGPT produced preliminary lists of highly and weakly expressed circRNAs, which were subsequently validated through literature review (Table 3). While ChatGPT serves as an accessible tool for beginners to understand basic workflows, DeepSeek offers deeper insights with experimental validation, making it more suitable for advanced research.

**Table 3:** Comparison of DeepSeek and ChatGPT in circRNA target identification for AML

Dimension	DeepSeek response	ChatGPT response	Key differences analysis
Data specificity	Provides specific circRNA IDs (e.g. hsa_circ_0004277) and experimental data (e.g. log2FC = 5.2, *P* = 1.3e−6)	Describes only analysis methods (e.g. DESeq2, EdgeR)	DeepSeek supports conclusions with real data, enhancing credibility
Mechanistic depth	Identifies ceRNA networks (e.g. hsa_circ_0004277/miR-20a-5p/BCL2L1 axis) and peptide-coding functions	Mentions only general concepts like ``sponging effect''	DeepSeek specifies molecular interactions, highlighting mechanistic innovation
Clinical translation	Proposes plasma biomarker panels (AUC = 0.93) and ASO therapy (IC50 = 18 nM)	Provides only generalized statements on ``potential therapeutic value''	DeepSeek quantifies translational indicators, directly guiding clinical application
Technological advancement	Integrates single-cell sequencing and CRISPR-dCas13 dynamic tracking	Does not cover cutting-edge technology applications	DeepSeek demonstrates technological integration potential, guiding future research directions

Abbreviations: CircRNA, Circular RNA; DESeq2, Differential Gene Expression Analysis Tool Based on Sequencing Data; EdgeR, Empirical Analysis of Digital Gene Expression Data From RNA-Seq Experiments; CeRNA, Competitive Endogenous RNA; MiR, MicroRNA; BCL2L1, BCL2-Like 1 Apoptosis Regulator; AUC, Area Under the Curve; ASO, Antisense Oligonucleotide; IC50, Half Maximal Inhibitory Concentration; CRISPR-dCas13, Clustered Regularly Interspaced Short Palindromic Repeats Dead Cas13 (Catalytically Inactive Cas13 Variant); Hsa_Circ_0004277, Human-Specific Circular RNA 0004277; Log2FC, Logarithm Base 2 Fold Change; P, Probability Value; MiR-20a-5p, MicroRNA-20a 5′ End Strand.

Ultimately, the optimal strategy for circRNA vaccine target screening involves integrating traditional bioinformatics tools for data validation, leveraging deep learning AI for precise predictions, and utilizing text-based AI for data mining and experimental optimization. This multimodal approach enhances both the efficiency and reliability of antigen screening in circRNA vaccine research.

### Inquiry into tumor antigen and immune cytokine sequences

In foundational tasks like gene sequence design, the accuracy of AI-driven tools has a direct impact on research and development efficiency. For instance, when prompted with the query “Provide the CDS sequence of IL-21”, ChatGPT tends to generate generalized explanations, often producing fictitious sequences (e.g*.* ATGGCCGAGG…) and referencing outdated sources, lacking practical validation methods for sequence accuracy. In contrast, DeepSeek leverages the latest NCBI database to retrieve the accurate RefSeq version of IL-21 (NM_021803.4), provides precise genomic coordinates (chr4:123,456–124,789), and includes experimental protocols like efetch command-line usage, thereby improving reproducibility (Table 4). This highlights the different adaptability levels of AI tools in scientific research. DeepSeek, with its emphasis on technical precision, is more suitable for experimental applications like molecular cloning, while ChatGPT is better for rapid conceptual overviews but lacks the accuracy required for high-precision research.

**Table 4:** Comparison of DeepSeek and ChatGPT in gene sequence design and accuracy for IL-21

Dimension	DeepSeek response	ChatGPT response	Key differences analysis
CDS length	Specifies 483 bp (encoding 160 AA)	Does not mention length or amino acid count	DeepSeek provides precise sequence length, while ChatGPT lacks specificity
Stop codon	Explicitly annotates TAA (positions 540–542)	Vaguely mentions ``possibly TAA, TAG, etc.''	DeepSeek provides exact stop codon location, ChatGPT remains uncertain
Retrieval method	Provides command-line tools (e.g. efetch) with examples	Only suggests manual NCBI website access	DeepSeek supports automated retrieval, ChatGPT lacks implementation details
Sequence accuracy	Links verified gene ID (59067) to NCBI	Uses placeholder symbols without real references	DeepSeek ensures sequence accuracy, ChatGPT lacks verifiable data
Potential errors	Identifies stop codon optimization and circularization site issues	Only mentions potential circRNA sequence conflicts	DeepSeek addresses specific design optimizations, ChatGPT remains general
Biological insights	Integrates experimental design details (e.g. vector selection, validation markers)	Limited to basic conceptual explanations	DeepSeek provides applied insights, ChatGPT remains theoretical
Tool recommendations	Suggests specialized tools (e.g. GeneDesign, OptimumGene)	No specific tool recommendations	DeepSeek provides actionable resources, ChatGPT lacks practical guidance

Abbreviations: CDS, Coding DNA Sequence; bp, Base Pair; AA, Amino Acid; TAA, Termination Codon; TAG, Termination Codon; NCBI, National Center for Biotechnology Information; eFetch, Entrez Programming Utilities eFetch Tool; GeneDesign, Gene Synthesis Optimization Software; OptimumGene, Codon Usage Bias Analysis Tool; circRNA, Circular RNA; StopCodon, Translation Termination Signal; Vector, DNA Delivery Vehicle; Validation Marker, Experimental Validation Marker.

### Synthetic design and optimization of CircRNA sequences

In the design of circRNA sequences, optimizing mRNA vaccine secondary structure and codon usage strategies significantly enhances mRNA stability against endonuclease degradation and chemical instability while improving translation efficiency and antibody response levels [78, 79]. Current sequence optimization uses an integrated, multi-dimensional approach to align biophysical principles with experimental data, maximizing efficiency. Codon optimization tools, such as GeneOptimizer and JCAT, utilize sliding window algorithms and host-specific parameters [e.g*.* tRNA abundance, GC content, Codon Adaptation Index (CAI)] to refine coding sequences. Additionally, these tools leverage rare codons to regulate ribosomal movement and facilitate proper protein folding [80, 81]. Concurrently, mRNA secondary structure prediction tools such as RNAfold and Mfold can identify and eliminate structural elements, such as hairpins or pseudoknots, which hinder translation efficiency. This "sequence-structure co-optimization" approach has been successfully applied in vaccine development [82, 83]. Recent progress suggests that hybrid architecture models trained on whole-genome datasets, such as Evo 2, have the potential to surpass conventional single-sequence optimization limitations. Its StripedHyena 2 framework, based on a convolution-Transformer hybrid network, utilizes a 1M-token context window to achieve synergistic optimization of codon usage bias, RNA secondary structure, and host genome interactions [77]. In addition, another algorithmic platform for circRNA, CircDesign, precisely regulates the spatial arrangement of the ORF and the IRES to effectively avoid structural conflicts between them. By employing the R3Design algorithm for tertiary structure prediction, it achieves a 44% correct folding rate and is capable of simultaneously optimizing the UTR regions, codon usage preference, and RNA secondary structure [84].

As mRNA vaccines encode antigens that must fold into functional proteins, optimizing protein structure prediction is equally crucial for enhancing immunogenicity. Traditional tools such as Rosetta and GROMACS rely on physical energy functions (e.g. van der Waals forces, hydrogen bonding) and molecular dynamics simulations. These methods maintain interpretability advantages in antigen epitope optimization (e.g. *CD19-*MHC complex modeling), but they are constrained by high computational costs (e.g. modeling proteins >200 amino acids) and the lack of dynamic information [Root Mean Square Deviation (RMSD) of 3–6 Å] [6, 85, 86]. In contrast, AI-driven tools such as AlphaFold 3 utilize deep neural networks to achieve sub-angstrom precision (1–2 Å), enabling predictions of protein-RNA/DNA interactions (e.g. *CD19*-chimeric antigen receptor t cell antibody complexes) and post-translational modifications (e.g. phosphorylation, glycosylation) [87].

While AI-driven tools have revolutionized protein structure modeling, generative AI models such as ChatGPT and DeepSeek extend these capabilities to RNA sequence design, offering new perspectives on structural optimization and codon refinement. For example, when tasked with designing a Group II intron-mediated PIE system for constructing and optimizing IL-21 circRNA plasmids, both models provided suggestions on framework design, core element selection, and validation workflows. However, discrepancies remain in sequence accuracy. DeepSeek generated precise sequence links and provided an in-depth optimization strategy from three perspectives: IL-21 cDNA refinement, enhancement of Group II intron splicing efficiency, and IRES and linker region design, incorporating directionality control, domain retention, and host interference suppression. In contrast, ChatGPT focused on macro-level strategic design but lacked key mechanistic insights (Table 5). Despite generative AI’s ability to foster strategy innovation, its black-box nature hinders biological rule internalization (e.g. codon-tRNA co-evolution patterns). Therefore, optimization outcomes still need to be validated using traditional computational tools.

**Table 5:** Comparison of ChatGPT and DeepSeek approaches in IL-21 circRNA sequence optimization

	ChatGPT approach	DeepSeek approach	Key differences summary
IL-21 cDNA optimization	Codon optimization	Uses mammalian high-expression codons, avoiding rare codons (e.g. AGG/AGA)	Optimizes codon usage based on human codon bias (Human Codon Usage Database), removes internal IRES-like sequences
GC content control	Not explicitly mentioned	Adjusts GC content to 45%–55%, avoiding high-GC regions (>70%)	DeepSeek defines specific GC range control
Hidden splice sites	Not mentioned	Uses SpliceAid/NetGene2 to predict and remove potential splice sites	DeepSeek emphasizes splice site interference risk control
Group II intron optimization	Splice site selection	Selects efficient splice sites (GUGA/UAA)	Preserves catalytic core domains (Domain V and VI), optimizes EBS/IBS complementary pairing (e.g. 5′-CAGUA-3′)
Directionality design	Not specified	Inserts 5′ intron in reverse and 3′ intron in forward orientation, enforcing reverse splicing	DeepSeek specifies directionality to enhance circularization efficiency
Host interference control	Not mentioned	Removes potential host microRNA binding sites within introns (predicted by miRBase)	DeepSeek emphasizes host RNA interference risk
IRES and linker optimization	IRES selection	Mentions IRES usage but does not specify type	Specifies EMCV IRES (GenBank: M81861.1) or HiPV IRES
Translation enhancement	Not specified	Adds Kozak sequence (GCCACCATGG) to optimize translation initiation	DeepSeek enhances translation via Kozak sequence
Linker design	Adds RBS-like enhancer sequence	Introduces Golden Gate cloning sites (e.g. BsaI/BsmBI) to ensure ORF remains in-frame post-splicing	Both optimize linker efficiency, but DeepSeek integrates cloning compatibility and ORF preservation

Abbreviations: IL-21, Interleukin-21 Cytokine; cDNA, Complementary DNA; Codon, Coding Triplet; GC, Guanine-Cytosine Content; Splice Sites, RNA Processing Sites; Group II Intron, Self-Splicing Non-Coding RNA Element; IRES, Internal Ribosome Entry Site; Translation, Protein Synthesis; Linker, Connecting Sequence; Golden Gate, Multiplex Cloning Technique; ORF, Open Reading Frame; Kozak, Translation Initiation Enhancer; miRBase, MicroRNA Registry Database; SpliceAid, Splice Site Prediction Tool; NetGene2, Neural Network-Based Splice Predictor; Human Codon Usage Database, Codon Frequency Reference; EMCV IRES, Encephalomyocarditis Virus Ribosome Entry Element; HiPV IRES, Hepatitis Delta Virus Ribosome Entry Element; BsaI/BsmBI, Type IIS Restriction Endonucleases; EBS/IBS, Exon/Intron Border Sequences; Domain V/VI, Catalytic RNA Domains; Rare Codons, Low-Frequency Codons; RBS-like Enhancer, Translation Enhancer Element; Reverse Splicing, Non-Conventional RNA Ligation; Catalytic Core, Enzymatic Functional Center; Sequence Fidelity, Genetic Accuracy; Host microRNA, Endogenous Regulatory miRNA; Cloning Compatibility, Vector Integration Efficiency; ORF Preservation, Coding Integrity Maintenance; Splice Junction Optimization, Enhanced RNA Processing; Eukaryotic Initiation, Translation Activation Process.

A future collaborative model integrating Rosetta’s energy-based optimization, AlphaFold 3’s structural predictions, and DeepSeek’s AI-driven sequence refinement can address both local sequence precision and global structural stability, enhancing the design of stable and translationally efficient circRNA molecules. This integrated strategy combines the strengths of traditional and AI-based tools, ensuring circRNA sequences are optimized for both translational efficiency and structural stability, while also supporting functional protein expression and immune response efficacy.

### Design of LNP delivery systems for CircRNA

Traditional bioinformatics tools such as PyMOL, NANOdesign, and POLYVIEW-3D support circRNA vaccine design through molecular dynamics simulations. PyMOL transforms LNP structures into three-dimensional dynamic models, visually representing mRNA encapsulation within the lipid bilayer and electrostatic interactions between SM-102 and the mRNA phosphate backbone [88]. Based on lipid composition models, NANOdesign predicts how varying SM-102/DSPC/cholesterol ratios affect encapsulation efficiency, although its predictions require experimental validation [89]. Meanwhile, POLYVIEW-3D simulates the dynamics of PEG-lipid membrane insertion, assessing LNP colloidal stability and particle size distribution [90]. These tools require manual input of parameters, such as buffer pH and temperature, and integration with cryo-EM data for model refinement, demanding expertise in both biology and computational chemistry.

In contrast, data-physics hybrid AI tools, such as TensorFlow, PyTorch, AI-guided Ionizable Lipid Engineering (AGILE) platform, leverage advanced algorithms to enhance circRNA vaccine design. TensorFlow, using CNNs and RNNs, analyzes LNP physicochemical properties and predicts how lipid molar ratios affect circRNA thermodynamic stability and delivery efficiency [91]. PyTorch’s dynamic computational graph enables real-time analysis of mRNA–LNP interactions in the immune microenvironment, optimizing antigen presentation pathways [92, 93]. AGILE applies deep learning to large-scale lipid datasets (>9000 structures) to predict ionizable lipid properties (e.g. apparent pKa, biodegradability) and design novel lipid structures. It can also predict the mRNA encapsulation efficiency, endosomal escape capability, and *in vivo* distribution of LNPs—e.g. enhancing lung targeting by adjusting the length or degree of unsaturation of hydrophobic tails [94].

LLM-driven platforms like DeepSeek and ChatGPT lower technical barriers through natural language interfaces, facilitating cross-disciplinary knowledge integration. When provided with the instruction “Design an LNP formulation targeting CD19 with an encapsulation efficiency >90%”, these models synthesize insights from nanomedicine and immunology, generating optimized lipid formulations (e.g. SM-102/DSPC/cholesterol = 50:10:38), microfluidic preparation parameters (e.g. flow rate ratio 3:1, buffer pH 4.0), and Python scripts for encapsulation efficiency analysis using RiboGreen® assays. Although these AI tools do not incorporate first-principles physical modeling, they derive statistical patterns from extensive literature and experimental data, enabling rapid prototyping, particularly useful in early-stage interdisciplinary research. Despite their value in early exploration, these models cannot replace experimental validation or the precision of traditional bioinformatics tools. Integrating AI with conventional methods will further accelerate mRNA vaccine design and support the precise development of personalized vaccines.

### Comparison between traditional and AI-driven approaches in vaccine development

Vaccine and immunotherapy development has traditionally relied on labor-intensive wet-lab techniques such as real-time quantitative reverse transcription PCR (qRT-PCR) and ELISpot assays. These methods require stepwise antigen screening, experimental immunogenicity validation, and iterative optimization, often spanning several months or even years. For example, the synthesis, purification, and delivery optimization of circRNA platforms often require multiple rounds of empirical testing [95]. In contrast, AI-driven approaches can significantly accelerate this process. Computational platforms enable rapid pre-screening of candidate antigens and in silico vaccine design, reducing the early discovery phase to just days or weeks. Deep learning models like MUNIS simulate epitope stability and binding affinity, replacing some experimental steps and accelerating T cell epitope identification [96, 97].

Traditional validation pipelines often exhibit low success rates and poor reproducibility. Techniques such as qRT-PCR are prone to variability, necessitating extensive screening to confirm the immunogenicity and safety of candidate antigens [98]. Studies have shown that only 30%–50% of screened candidates pass *in vitro* immunogenicity assays [99]. In contrast, AI-assisted bioinformatics tools can more efficiently prioritize high-potential candidates. For instance, AI-based predictions of MHC class II epitopes have achieved immunogenicity accuracy rates of 70%–80%, significantly reducing ineffective experimental efforts [100].

Candidate antigen coverage is another critical point of divergence. Traditional methods, limited in throughput, often target a single antigen or epitope, leading to suboptimal population coverage [101]. AI-based strategies, however, enable comprehensive epitope profiling and multi-epitope vaccine design, optimized for broad population coverage. Computational algorithms simulate diverse HLA allele distributions, enabling cross-ethnic applicability, with theoretical MHC-I and MHC-II coverage reaching 98.29% and 81.81%, respectively [102, 103].

Conventional immunogenicity prediction relies heavily on animal models or *in vitro* assays such as enzyme-linked immunosorbent assay (ELISA) and intracellular cytokine staining (ICS), both of which are time-consuming and costly [104, 105]. For example, mRNA vaccine efficacy often undergoes multi-step evaluation using hemagglutination inhibition assays [104]. AI models combine molecular docking with deep learning to predict antigen–TLR4 interactions or T cell responsiveness, with high concordance between computational and experimental results [97].

Finally, personalized vaccine design remains a major challenge in traditional paradigms due to the high cost and complexity associated with individualized sequencing and manufacturing, as exemplified by clinical trials for glioblastoma (GBM) vaccines [106]. AI-driven pipelines rapidly generate personalized antigen libraries from patient-specific tumor sequencing data [105]. Beyond neoantigen selection, AI can optimize multiple elements of circRNA constructs—including IRES selection and codon usage—to enhance expression and translational efficiency [107]. These capabilities support the design of tailored multi-epitope vaccines for specific cancers, enhancing the scalability and clinical feasibility of personalized immunotherapy [108]. A summary of the key differences is provided in Table 6.

**Table 6:** Comparison of traditional and AI-driven approaches in circRNA vaccine development

Dimension	Traditional experimental approaches	AI-driven approaches
Development timeline	Prolonged (typically years), dependent on iterative experimental optimization	Accelerated (weeks to months), facilitated by computational pre-screening and modeling
Validation efficiency	Relatively low success rate (30%–50%) with limited reproducibility	Higher predictive accuracy (70%–80%), significantly reducing ineffective experiments
Candidate diversity	Narrow scope (typically single antigen or epitope)	Broad coverage (multi-epitope combinations, population-level immunogenicity optimization)
Immunogenicity prediction	Predominantly reliant on *in vivo* or *in vitro* assays, associated with high cost and time burden	Prioritized through in silico modeling, followed by targeted experimental validation
Personalization potential	Limited by high costs and scalability challenges	Highly adaptable with low cost, enabling rapid design of personalized vaccines (e.g. neoantigen-based)

Abbreviations: *in vivo*, Within a Living Organism; *in vitro*, In an Artificial Laboratory Environment; *in silico*, Performed via Computer Simulation; *e.g*., For Example; Development Timeline, Time Required to Complete a Process; Validation Efficiency, Reliability of Experimental Confirmation; Candidate Diversity, Breadth of Potential Target Options; Immunogenicity Prediction, Likelihood of Inducing an Immune Response; Personalization Potential, Capacity for Individualized Adaptation; Success Rate, Proportion of Successful Outcomes; Reproducibility, Consistency of Experimental Results; Predictive Accuracy, Precision of Computational Predictions; Epitope, Specific Antigenic Determinant; Neoantigen, Newly Formed Antigenic Target; Population-Level, Applicable Across Broad Demographic Groups; *in vivo* Assay, Testing Conducted in Living Systems; *in vitro* Assay, Testing Conducted in Controlled Laboratory Environments; *in silico* Modeling, Simulation-Based Computational Modeling; Multi-Epitope, Involving Multiple Antigenic Determinants.

## Discussion and Conclusion

mRNA vaccines have exhibited remarkable potential in the prevention of infectious diseases and cancer immunotherapy owing to their rapid design, high expression efficiency, and flexible optimization. However, their clinical application is constrained by susceptibility to enzymatic degradation and the induction of innate immune responses. In contrast, circRNA vaccines, with their covalently closed-loop structure, offer enhanced stability and reduced immunogenicity, rendering them promising candidates for next-generation vaccines. Despite this, challenges such as low circularization efficiency and poor compatibility with delivery systems remain significant barriers to clinical translation.

To address these challenges, researchers have begun integrating the complementary advantages of mRNA and circRNA platforms, while also incorporating AI technologies to accelerate iterative vaccine design. Bioinformatics AI has already achieved breakthroughs in antigen prediction, sequence optimization, and delivery system modeling. Compared to conventional database-driven methods, such as NetMHC [74] and IEDB-AR [75], deep learning models, such as DeepBind [76] and Evo 2 [77], utilize CNNs and Transformer architectures to extract critical antigenic epitopes from multi-omics data, significantly improving prediction accuracy. Furthermore, AI can enhance RNA stability and translation efficiency by optimizing codon usage, RNA secondary structure, and host compatibility, and can also improve antigen design by modeling RNA-protein interactions. In delivery system optimization, bioinformatics AI platforms, such as TensorFlow [109], can simulate the impact of LNP components on mRNA stability and encapsulation efficiency, offering superior dynamic modeling capabilities over traditional molecular dynamics tools such as PyMOL [88] and POLYVIEW-3D [90].

Generative AI, on the other hand, demonstrates unique advantages in literature summarization, experimental design, and data analysis. For instance, in this study, DeepSeek has shown strong literature support and experimental feasibility for circRNA target prediction, while ChatGPT is more suitable for foundational analytical workflows. Additionally, these models can generate antigen target analysis reports by integrating existing databases, recommend experimental protocols, and even recommend LNP compositions and related experimental conditions, thereby accelerating research workflows. However, generative AI may produce hallucinated citations during literature retrieval, lacks a deep understanding of biological constraints in sequence design, and offers limited reliability in experimental parameter suggestions. Consequently, AI-generated content must be validated using traditional bioinformatics tools and laboratory experiments to ensure scientific rigor and real-world applicability.

While AI provides powerful tools for predicting circRNA vaccine properties, such as immunogenicity, stability, and delivery efficiency, these in silico predictions must be rigorously validated through biological experimentation. For instance, although experimental data have demonstrated that circRNAs possess significantly longer *in vivo* half-lives than linear mRNAs [110], the efficiency of various circularization strategies, such as enzymatic ligation and ribozyme-mediated self-splicing, differs considerably and requires empirical evaluation [111, 112]. Moreover, while AI models predict reduced innate immune activation by circRNAs, preclinical studies have shown that combining circRNA vaccines with immune checkpoint inhibitors can enhance antitumor responses in murine models [113], indicating that immunoregulatory mechanisms may be more complex than currently understood.

To evaluate vaccine efficacy, the ability of circRNA constructs to induce neutralizing antibodies, such as those encoding monkeypox virus surface antigens, must be quantitatively assessed using ELISA or similar immunoassays [114]. Moreover, although LNPs are effective for delivering mRNA, the larger molecular size and unique structural properties of circRNAs may hinder encapsulation efficiency. This necessitates *in vivo* validation through methods such as fluorescence labeling or qPCR to assess biodistribution and targeting specificity [115, 116].

AI can also facilitate the rational design of novel circularization enzymes and chemical modification strategies to enhance circRNA production [117]. However, experimental validation remains critical to detect unintended effects, such as the formation of immunogenic dsRNA byproducts [118]. Ultimately, AI-optimized circRNA vaccine candidates must be evaluated in non-human primate models to accurately assess their immunogenicity and translational potential [114, 119]. These examples highlight the indispensable role of biological validation in refining AI-driven vaccine design and underscore the importance of a synergistic approach to precision vaccinology [114]. Furthermore, although LNPs are effective for mRNA delivery, the larger molecular size and distinct structural characteristics of circRNAs may compromise their encapsulation efficiency. This limitation necessitates *in vivo* validation, such as through fluorescence labeling or qPCR, to evaluate biodistribution and targeting specificity [115, 116].

AI can also contribute to the rational design of novel circularization enzymes and chemical modification strategies to improve circRNA production [117]. Nevertheless, experimental validation remains indispensable to detect unintended consequences, such as the formation of immunogenic dsRNA byproducts [118]. Ultimately, AI-optimized circRNA vaccine candidates must be evaluated in non-human primate models to accurately assess their immunogenicity and translational potential [114, 119]. These examples underscore the indispensable role of biological validation in refining AI-guided vaccine design and enabling a synergistic approach to precision vaccinology.

In summary, despite the transformative potential of AI in vaccine research, its application in high-precision biomedical settings remains constrained by several factors. First, the “black-box” nature of deep learning models reduces interpretability, making it challenging to understand the rationale behind predictions—an issue particularly limiting for mechanistic studies and regulatory decision-making, where transparency is paramount. Second, the performance of AI models heavily depends on the quality and representativeness of training data. Biased datasets can lead to skewed predictions and poor generalizability. To mitigate these limitations, integrating heterogeneous data sources, such as immunological response profiles across diverse populations, and adopting standardized formats like the observational medical outcomes partnership (OMOP) Common Data Model can help reduce sampling bias [120]. For example, vaccine design targeting ESKAPE pathogens requires training data encompassing diverse genetic and geographic pathogen variants [121]. Techniques such as adversarial training or sample reweighting can further address data imbalance, particularly underrepresented immunogenicity data in public datasets [122]. The test-negative design is also effective for reducing bias in vaccine efficacy assessments caused by healthcare-seeking behavior [123].

For improved interpretability, linear models or attention-based frameworks—such as the ISA-PN model—are preferred, as they quantify the contributions of molecular substructures through positive and negative attention scores and thereby achieve a balance between accuracy and transparency [124]. For epitope prediction, methods like SHAP can elucidate the decision-making process of long short-term memory (LSTM)-based models [125], while visualization tools such as U-AnoGAN and factor regression can highlight anomalous regions or explain latent features, thereby improving clinician trust [126]. However, AI-generated literature reviews may contain outdated or hallucinated content, potentially compromising research integrity. Moreover, AI-based designs for biological sequences or experimental conditions remain inherently imperfect, necessitating empirical verification. Thus, while AI can streamline experimental planning, it must be tightly integrated with traditional computational biology and validated through biological experimentation to ensure reliability.

The development of circRNA vaccines also faces significant regulatory hurdles. These include evaluating potential off-target effects and aberrant immune activation, uncertainties regarding the long-term impact of circRNA structures on cellular RNA metabolism [26, 127], and biosafety concerns associated with AI-generated sequences. Manufacturing and QC present additional complexities: AI-optimized synthesis protocols require rigorous standardization; low circularization efficiency may introduce batch variability; and residual linear RNA or enzymatic impurities can hinder quality assurance [119, 128]. Existing LNP delivery platforms lack sufficient tissue specificity and pose hepatotoxicity risks [119]. Most preclinical data derive from animal models, with limited validation in humans [129] and insufficient long-term follow-up [108]. Clinical trials must also address ethical considerations related to AI training on large datasets, requiring both data representativeness and compliance with data privacy regulations [130].

To facilitate clinical translation, expedited regulatory pathways specific to AI-enabled technologies should be developed, along with circRNA-specific guidance informed by mRNA vaccine experiences, particularly regarding stability and translational efficiency. International cooperation is crucial for harmonizing QC standards, and academia–industry partnerships are needed to promote data sharing for AI validation. On the innovation front, new *in vitro* and *in vivo* models are required to evaluate AI-designed circRNA vaccines, while AI itself should be employed to further optimize immunogenicity and delivery. Real-world data should also be used for long-term safety monitoring, with stratified evaluations for multivalent vaccines to assess potential component interactions. Ultimately, regulators must balance innovation with risk to enable the safe and effective transition of AI-designed circRNA vaccines from laboratory to clinic. With continued advancements in AI, key challenges such as delivery efficiency, stability, and immunogenicity are expected to improve, but corresponding ethical and global regulatory frameworks must evolve in parallel.

The application of AI in biomedical research also presents several ethical challenges. First, the “black-box” characteristics of AI systems limit transparency and hinder clinical trust, necessitating the development of explainable AI (XAI) aligned with existing frameworks such as idea, development, exploration, assessment, long-term follow-up study (IDEAL) and standardized reporting of machine learning applications in urology (STREAM-URO) [131]. Second, AI may amplify pre-existing data biases, potentially exacerbating health disparities among underrepresented populations. This calls for the inclusion of diverse datasets and algorithm audits to promote fairness [132]. The use of sensitive health data also heightens the risk of privacy breaches, requiring robust de-identification, encryption technologies, and adaptive informed consent mechanisms [132]. While regulations such as the general data protection regulation (GDPR) mandate clear data usage policies, existing consent models are ill-suited to the dynamic and iterative nature of AI-driven research [133]. Moreover, accountability for AI-generated errors remains ambiguous, necessitating clearer frameworks to delineate responsibilities among developers, researchers, and clinicians. In the context of generative AI tools such as ChatGPT, academic integrity standards must also be established to ensure responsible use in scientific writing and discovery [134].

**Key Points**

- Circular RNA vaccines offer enhanced stability and reduced immunogenicity compared to linear mRNA vaccines, making them a promising platform for cancer immunotherapy and infectious disease prevention.
- Bioinformatics AI, particularly deep learning models like convolutional neural networks, improves antigen prediction, RNA structure modeling, and lipid nanoparticle delivery system optimization, surpassing traditional bioinformatics methods.
- Generative AI accelerates literature synthesis and experimental planning but faces challenges such as reference fabrication and limited biological interpretability, necessitating careful validation.
- Combining AI-driven tools with traditional bioinformatics and experimental validation enhances vaccine development, ensuring both computational efficiency and biological reliability.
- Advancing interpretable AI models, integrating real-time experimental feedback, and establishing ethical AI frameworks will be crucial for improving precision vaccine development and clinical applications.
